# Chaihu-Longgu-Muli decoction improves sleep disorders by restoring orexin-A function in CKD mice

**DOI:** 10.3389/fendo.2023.1206353

**Published:** 2023-06-27

**Authors:** Xin-li Cao, Xue-mei Peng, Gong-bo Li, Wei-sen Ding, Kai-zhen Wang, Xiao-lei Wang, Yan-ying Xiong, Wei-jian Xiong, Fan Li, Min Song

**Affiliations:** ^1^ Department of Nephrology, Chongqing Hospital of Traditional Chinese Medicine, Chongqing, China; ^2^ Department of Traditional Chinese Medicine, Chongqing General Hospital, Chongqing, China; ^3^ Department of Neurology, Second Affiliated Hospital of Chongqing Medical University, Chongqing, China

**Keywords:** Chaihu-Longgu-Muli decoction (CLMD), chronic kidney disease (CKD), insomnia, orexin-A, inflammation, metabolic disorders

## Abstract

**Introduction:**

Chaihu-Longgu-Muli decoction (CLMD) is a well-used ancient formula originally recorded in the “Treatise on Febrile Diseases” written by the founding theorist of Traditional Chinese Medicine, Doctor Zhang Zhongjing. While it has been used extensively as a therapeutic treatment for neuropsychiatric disorders, such as insomnia, anxiety and dementia, its mechanisms remain unclear.

**Methods:**

In order to analyze the therapeutic mechanism of CLMD in chronic renal failure and insomnia, An adenine diet-induced chronic kidney disease (CKD) model was established in mice, Furthermore, we analyzed the impact of CLMD on sleep behavior and cognitive function in CKD mice, as well as the production of insomnia related regulatory proteins and inflammatory factors.

**Results:**

CLMD significantly improved circadian rhythm and sleep disturbance in CKD mice. The insomnia related regulatory proteins, Orexin, Orexin R1, and Orexin R2 in the hypothalamus of CKD mice decreased significantly, while Orexin and its receptors increased remarkably after CLMD intervention. Following administration of CLMD, reduced neuron loss and improved learning as well as memory ability were observed in CKD mice. And CLMD intervention effectively improved the chronic inflflammatory state of CKD mice.

**Discussion:**

Our results showed that CLMD could improve sleep and cognitive levels in CKD mice. The mechanism may be related to the up-regulation of Orexin-A and increased phosphorylation level of CaMKK2/AMPK, which further inhibits NF-κB downstream signaling pathways, thereby improving the disordered inflammatory state in the central and peripheral system. However, More research is required to confirm the clinical significance of the study.

## 1 Introduction

Chronic kidney disease (CKD) refers to the reduction of glomerular filtration rate and irreversible damage to renal structure as well as function due to various causes (1). Compared with patients with other chronic diseases, CKD patients often require longer and more frequent hospitalizations. As the disease progresses, the incidence of metabolic syndrome is significantly higher than that of the general population. Therefore, the goal of CKD treatment includes not only delaying the progression of the disease, but also reducing complications and accompanying symptoms.

CKD patients often experience sleep problems such as broken sleep, short sleep time, and poor sleep quality (2, 3). Some CKD patients even experience varying degrees of memory decline. It is currently believed that the decrease in sleep efficiency in CKD patients is related to decreased hemoglobin, blood phosphorus, and serum urea nitrogen levels. In addition, complement activation in CKD patients stimulates monocytes to produce interleukin-1, interleukin-6, and tumor necrosis factor(4, 5). An increase in core body temperature activates the cooling mechanism, thereby enhancing daytime sleep tendencies and leading to a decrease in night-time sleep (6). Therefore, there is an urgent need to develop drugs to improve metabolic disorders in CKD patients while improving their sleep and quality of life.

The Chaihu-Longgu-Muli decoction (CLMD) was originally recorded in the “Treatise on Febrile Diseases” written by the founding theorist of Traditional Chinese Medicine, Doctor Zhang Zhongjing, 1,800 years ago. CLMD has been used extensively as a therapeutic treatment for neuropsychiatric disorders, such as insomnia, depression, anxiety, and dementia(7–9), but its mechanism is still unclear.

Awakening and sleep are mainly controlled by glutamate and γ-Aminobutyric acid(GABA) neurons through local as well as distant projections and interactions, where a signal regulation system composed of neurotransmitters regulates cortical activities and behaviors in the wake/sleep state. Orexin is an important neurotransmitter and plays a major role in stabilizing arousal (10). As a neuropeptide produced by the hypothalamus, orexin binds to orexin receptors on the surface of the nucleus neurons involved in sleep regulation to act as a “trigger switch” for sleep and wakefulness, while maintaining the coordination and stability of the transition period (11). Previous studies have shown that the expression of orexin-A and orexin receptor-1 in the hypothalamus of CKD rats is low (12), indicating that orexin-A might be a therapeutic target of CKD-related insomnia.

In this experiment, we evaluate the effects of CLMD in insomnia and explore the molecular mechanisms. After an adenine diet-induced CKD mouse model was established, insomnia-related behaviors were observed after treatment with CLMD. We aimed to observe the potential neurobiological mechanisms by observing the orexin-A-related signal pathway in the hypothalamus and hippocampus and effect of treatment with CLMD.

## 2 Materials and methods

### 2.1 Animals

Specific pathogen-free(SPF)male C57/BL6 mice, weighing between 25–27g, were raised in the Experimental Animal Center of Chongqing Hospital of Traditional Chinese Medicine. The environmental temperature was set at 24 ± 2°C with a relative humidity of 60 ± 5%, and the mice ate and drank freely in alternating light and dark for 12 hours. The animal experiment methods were based on the Guidelines for the Care and Use of Laboratory Animals and were approved by the Animal Care and Use Institutional Committee of Chongqing Hospital of Traditional Chinese Medicine(NO.2020-DWSY-LF).

### 2.2 Drugs

The CLMD formula contained the following: *Bupleurum chinense DC.* (12 g), *Fossilia Ossia Mastodi*(4.5 g), *Scutellaria baicalensis Georgi*(4.5 g), *Zingiber officinale Roscoe* (4.5 g), *Panax ginseng C. A. Mey.* (4.5 g), *Cinnamomum cassia* (L.) *J.Presl* (4.5 g), *Poriacocos (Schw.) Wolf* (4.5* g*), *Pinellia ternata* (Thunb.) *Makino* (6g), *Rheum palmatum L.* (6* g*), *Ostrea gigas Thunberg* (4.5 g), and *Ziziphus jujuba Mill.* (4.5* g*). All drugs were purchased from the Pharmacy Department of Chongqing Hospital of Traditional Chinese Medicine.

**Figure 1:**
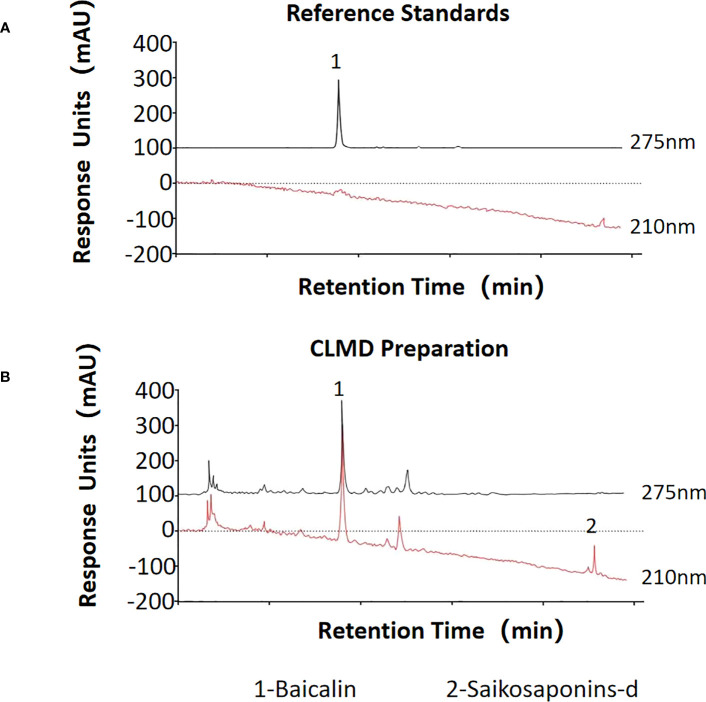
The quantities of two characteristic pharmaceutically active substances in CLMD preparation for study. **(A)** Reference standards; **(B)** CLMD preparation. CLMD: Chaihu-Longgu-Muli Decoction.

To ensure the consistency of this formula, the quality of each component was checked, and the extraction of the decoction strictly followed the standard procedures described in the Pharmacopoeia of the People’s Republic of China (26). The formula was were soaked in water for 30 minutes, and then decocted with boiling water at a ratio of 1:8 for 2 h, subsequently concentrated to make a paste, containing 1 g of crude extracts per gram. The paste was diluted to a solution with the concentration of 1 g/ml by distilled water, which was stored at 4°C for further use. The contents of two characteristic pharmaceutically active components of CLMD, saikosaponins-d and baicalin, were measured using high performance liquid chromatography (HPLC) (**Figure 1**). The HPLC result of the CLMD solution was consistent with those in previous studies (13).

### 2.3 Model establishment and experimental design

#### 2.3.1 Establishment of mice chronic renal disease model

Previous studies have shown that administering an adenine diet can lead to chronic kidney damage, such as massive proteinuria, deterioration of renal function, and pathological damage of renal tubules and interstitial tissues. Therefore, this study followed the literature (14).

After 60 C57/BL6 mice were fed with a 0.2% adenine diet for 2 weeks, four mice were selected for detection of serum creatinine and urea nitrogen indicators, and 50 mice were randomly selected to be weighed and enter the follow-up experiment.

#### 2.3.2 Establishment of experimental groups

Purine-fed mice were randomly divided into CKD(n=10), CLMD-Low dose group(CLMD-L,n=10), CLMD-Medium dose group(CLMD-M, n=10), and CLMD-High dose group(CLMD-H, n=10), and 10 normal diet mice comprised a control group. The CKD, CLMD-L, CLMD-M, and CLMD-H groups continued the purine diet, while the CLMD-L, CLMD-M, and CLMD-H groups were also given CLMD by gavage at a dose of 2.36 g/kg, 4.725 g/kg, and 9.45 g/kg, respectively, and the CKD group was given a normal saline gavage. The control group continued the normal diet and were administered saline for 4 weeks. 24-hour spontaneous activity monitoring was carried out 3 days before the end of the gavage to evaluate circadian rhythms. After completion of the gavage, mice were weighed and then performed a 6-day water maze behavior test.

Following this, four of the mice were deeply anesthetized and perfused transcardially with cold PBS followed by 4% paraformaldehyde and processed for histochemical and immunohistochemical analyses. Six of the mice were euthanized and their eyeballs and fresh brain tissue samples were collected for enzyme-linked immunosorbent assay (ELISA) and western blotting (WB) detection.

### 2.4 Analysis of spontaneous activities

The circadian rhythms of the mice were monitored using a spontaneous activity experiment video analyzer set to a 12D/12L light-dark cycle (0–12 h was light-on time, and 12–24 h was light-off time), and the mouse activity was recorded every 2 h. Each recording lasted for 3 minutes, during which the length of time and the number of activities performed by the mice was recorded. Activities were recorded over a 3-day period, and the difference between the longest and shortest time of activity in each group of mice (recorded as time variability) was compared. Changes in mouse circadian rhythms were also analyzed.

### 2.5 Pentobarbital-induced sleep behavior record

Dilute pentobarbital with 0.9% normal saline to 1%, experimental mice was injected intraperitoneally at a dose of 45mg/kg (15, 16). The time from pentobarbital injection to the disappearance of the righting reflex was recorded as the sleep latency. The time from the beginning of sleep to the disappearance of the righting reflex was recorded as the sleep time. The sleep latency and sleep time of mice in each group were compared.

### 2.6 Morris water maze assessment of mouse learning and memory

After the gavage, the Morris water maze (MWM) was used to test the spatial learning and memory abilities of the mice in each group. Five-day memory training was undertaken. The mice were placed in a circular pool with three quadrants. An underwater platform was placed in the third quadrant, and the mice were placed in the opposite quadrant. The mice were placed in the water and the time taken for the mouse to reach the underwater platform was monitored (escape latency, within 90s). If the mouse did not find the platform within 90s, it was guided to the platform and made to stay there for 30s. After training for 5 consecutive days, a space exploration experiment was conducted on the 6th day after the training. The mouse exploration trajectory was recorded within 90s, and the number of platform crossings during that time was recorded, as well as the percentage of time spent in the target quadrant.

### 2.7 ELISA kit index detection

Blood was collected from mouse eyeballs, subsequently incubated for 1 hour and then centrifuged at 4000 rpm for 15 minutes at low temperature. The supernatant was collected for further analysis. ELISA kits were used to measure levels of the following inflammatory factors: serum creatinine (C011-1-1; Nanjing Jiancheng, Bioengineering Institute, Nanjing, China), urea nitrogen (C013-1-1; Nanjing Jiancheng, Bioengineering Institute), peripheral tumor necrosis factor-alpha (TNF-α)

(H052; Nanjing Jiancheng, Bioengineering Institute), interleukin-6 (IL-6)(H007; Nanjing Jiancheng, Bioengineering Institute), and IL-1β (H002; Nanjing Jiancheng, Bioengineering Institute). Fresh brain tissue was also collected from the hippocampal protein homogenate and passed through a bicinchoninic acid (BCA) assay kit (H004; Nanjing Jiancheng, Bioengineering Institute) to measure the protein concentration. Following normalization to total protein, IL-10 and IL-4 expression levels were quantified.

### 2.8 Western blot detection of protein expression level

The expression levels of orexin, orexin receptor1, and orexin receptor2 in the mouse hypothalamus, as well as NF-κB, CaMKKII, AMPK and their phosphorylated proteins derivatives in the hippocampus were measured. The protein expression levels of TNF-α and IL-1β in the hippocampus were also detected. The mouse hippocampus tissue protein was extracted according to the experimental procedure. The concentration of each sample was calculated using BCA. After normalization, 20 μg of the sample was placed into each well. After gel electrophoresis, the membrane was transferred and blocked at room temperature for 2h. The primary antibody was diluted in different proportions at 4°C overnight, washed with triton-X-100 TBS(TBST) (1:10000) for 3×5 min. It was then incubated for 2 h at room temperature, washed with TBST for 3×10 min, and placed in ECL luminescent solution to visualize the band. Image J 1.41 software was used to measure the mean gray value of the bands and applied for statistical analysis. Primary antibodies used were: orexin (ab255294; Abcam, Cambridge, MA, USA), orexin R1 (ab77370; Abcam), orexin R2 (PA5-77567; Thermo Fisher Scientific, Lafayette, CO, USA), TNF-α (11948S; Cell Signaling Technology [CST], Danvers, MA, USA); IL-1β (31202S; CST), p-NF-κB P65 (3033S; CST); NF-κB (8242S; CST), CaMKK2 (16810s; CST), phospho-CaMKK2 (Ser511) (12818; CST), AMPK (2532S; CST); and p-AMPK (2535S; CST). Secondary antibodies used were: goat anti- mouse IgG(H+L) (1:10000) (115-005-003; Jackson ImmunoResearch Laboratories Inc., West Grove, PA, USA) and goat anti-Rabbit IgG(H+L) (1:10000) (111-005-003; Jackson ImmunoResearch Laboratories Inc.).

### 2.9 Immunofluorescence staining

After the mice were perfused with paraformaldehyde, the whole brain was taken, fixed, dehydrated, and then frozen sectioned with a thickness of 20 μm. Next, the tissue was washed twice with PBS, permeabilized with 0.5% Triton for 40 minutes, blocked with 5% bovine serum albumin(BSA) for 1 h, placed in 1:400 anti-NeuN (26975-1-AP; ProteinTech Group Inc., Rosemount, IL, USA) overnight at 4°C, and washed with PBS three times. Secondary donkey anti-rabbit antibody-AF555 (6441-32; SouthernBiotech, Birmingham, AL, USA) was added at 1:500 and samples were left for 2 h at room temperature, then washed twice with PBS, placed in DAPI for 20 min, mounted on a slide, observed under a fluorescence microscope. The NeuN positive cells were counted (where four mice in each group were selected for frozen section, three sections were taken for each mouse, and hippocampus CA1 area as well as CA3 area were selected for cell count).

### 2.10 Statistical analysis

SPSS19.0 software (IBM Corp., Armonk, NY, USA) was used for statistical analysis. Student’s t-test or one-way ANOVA were used to analyze the significant differences in groups. Data are presented as the Mean ± Standard error of the mean (SEM). In all cases, P < 0.05 was considered statistically significant.

## 3 Results

### 3.1 CLMD improved circadian rhythms and sleep state in CKD mice

**Figure 2:**
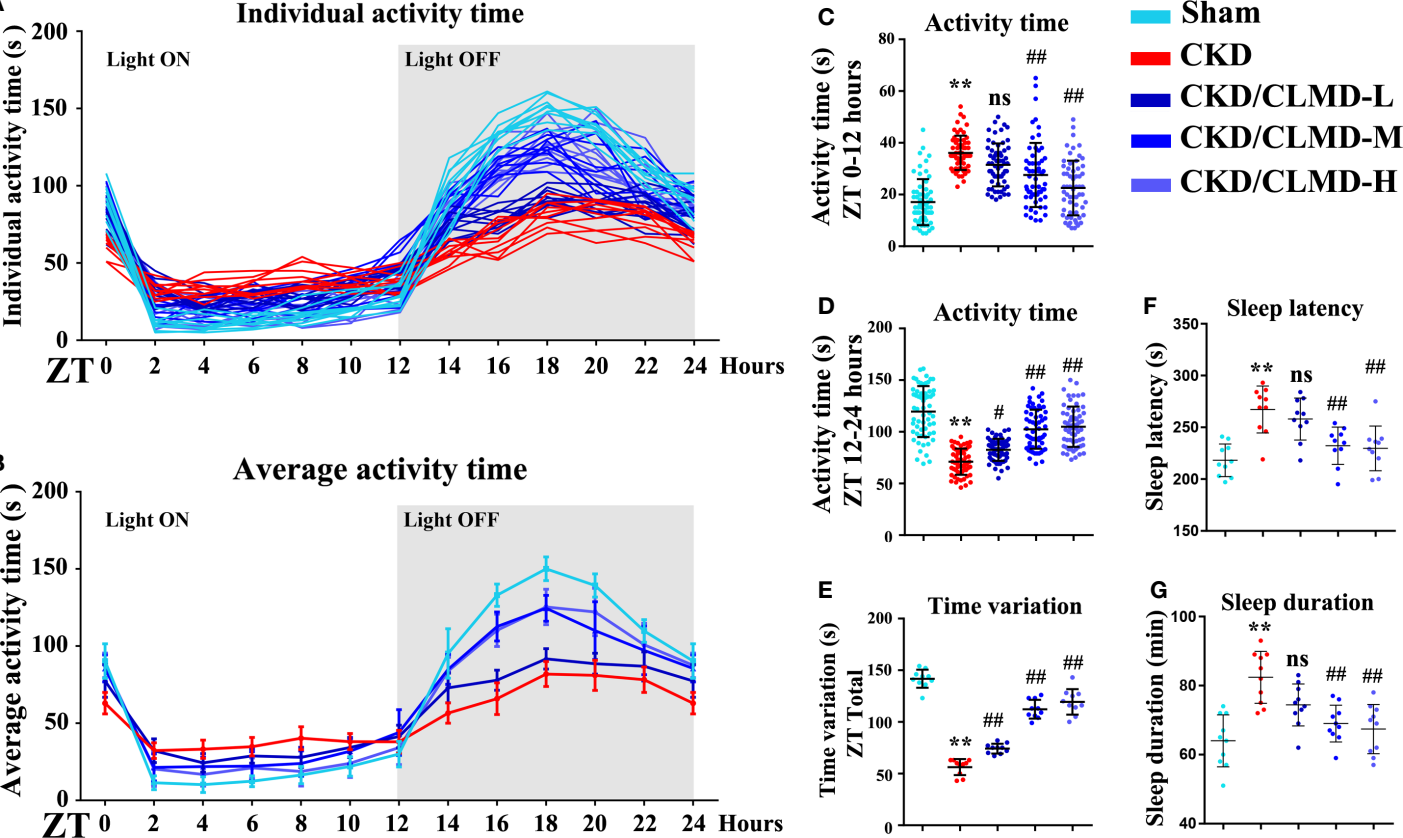
Cage activity following CLMD treatment among adenine-induced chronic kidney disease (CKD) mice. After 4 weeks of control or CLMD treatment in CKD mice (n=10 in each group), cage activity **(A–E)** was measured by infrared sensor and video monitoring. Time-series activity data are shown with individual plots **(A)**; 2-hour bins **(A, B)**; and 12-hour bins **(C, D)**. Time variations are shown as the difference between the highest and lowest activity time of each mouse **(E)**. The dark shadows indicate the time of lights-off (Zeitgeber time; ZT0 is the time of light-on and ZT12 is the time of light-off; ZT12–24). Effects of CLMD on hypnotic response in CKD mice in the pentobarbital-induced sleep test—sleep latency **(F)** and sleep duration **(G)**—are shown. All values are expressed as individual plots with mean ± standard deviation. P values versus control: *P < 0.05; **P < 0.01; P values versus CKD: #P < 0.05; ##P < 0.01; ns P > 0.05.

In order to explore the effect of CLMD on sleep disorders in CKD mice, we used pentobarbital sleep induction and monitored mouse activity for 24h. The results showed that in spontaneous activity monitoring for 3 consecutive days, compared with the control group, CKD mice showed reduced activity during the light-off time and increased activity time during the light-on time. The rhythm of the activity curve was poor, and the 24-hour activity variability was significantly reduced after intervention with CLMD. The low-dose group did not show any significant changes, but with an increase in dose, the number of light-on activities of mice in the CLMD-M and CLMD-H groups decreased, and the number of activities increased after the light was turned off, which differed from the control group pattern. The trend was gradually approaching significance, where the overall results suggest that CKD mice lack a clear circadian rhythm. In the pentobarbital sleep induction experiment, CKD mice showed increased sleep latency and increased sleep time, indicating that CKD mice had sleep disorders (in this specific case induced by pentobarbital). Overall, the time for CKD mice to fall asleep increased. However, due to the decreased metabolic function of the kidneys in mice, the rate of drug metabolism decreased, and the time required to recover wakefulness increased after intervention with CLMD. As the drug dose increased, the sleep latency and sleep time of CKD mice were gradually shortened. Taken together, the above two findings indicate that CKD mice show circadian rhythm disturbances and sleep disturbances and CLMD can improve this phenomenon. The above results are shown in **Figure 2**.

### 3.2 CLMD improved the learning and memory ability of CKD mice

**Figure 3:**
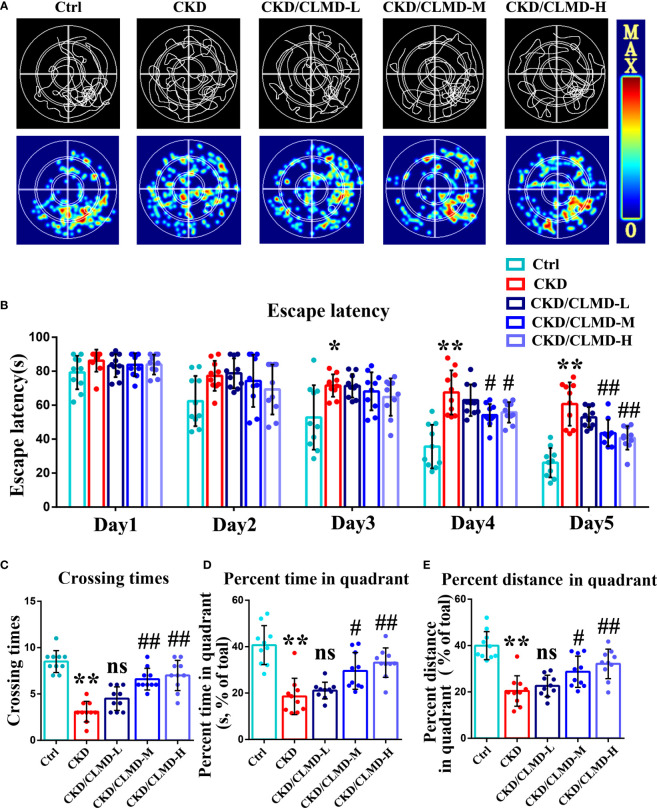
Representative swimming traces and heat map of different CKD mice groups in the MWM test **(A)**. The water maze area was defined by four quadrants. The small circle in quadrant 4 was the platform position. Quantitative analysis of the time taken to find the platform in the MWM test **(B)**. The number of times a mouse crosses the platform **(C)**, the percent time spent **(D)**, and the percent distance **(E)** in the quadrant area in the spatial probe test are shown. The data are presented as mean ± standard deviation (n = 10/group). All values are expressed as individual plots with mean ± standard deviation. P values versus control: *P < 0.05; **P < 0.01; P values versus CKD: #P < 0.05; ##P < 0.01; ns P > 0.05.

To further determine the effect of CLMD on learning and memory in CKD mice, after improved sleep, we used the MWM (a hippocampus dependent task) to analyze the effect of CLMD on spatial memory and learning ability. MWM requires multiple days of training and is considered the gold standard for quantification of spatial memory (17). The results showed that the escape latency of mice in each group was gradually shortened on the 1st to 5th day of navigation in the water maze, compared with that of the control group in the 3rd, 4th, and 5th day of the experiment, and the escape latency of the model group was prolonged (P<0.05, P<0.01). On the 4th and 5th days of training, compared with that of the CKD group, the escape latency of the CLMD medium and high-dose groups was shortened (P<0.05, P<0.01). The space exploration experiment showed that, compared with that in the control group, the number of platform crossings, the target quadrant stay time, and the proportion of distance in the CKD group were significantly reduced (P<0.01). The CLMD dose groups increased the number of platform crossings in CKD mice and the target quadrant stay time, as well as the proportion of time and distance. The above results indicate that CLMD can improve the learning and memory ability of CKD mice. These results are shown in **Figure 3**.

### 3.3 CLMD significantly reduced neuron loss in CKD mice

**Figure 4:**
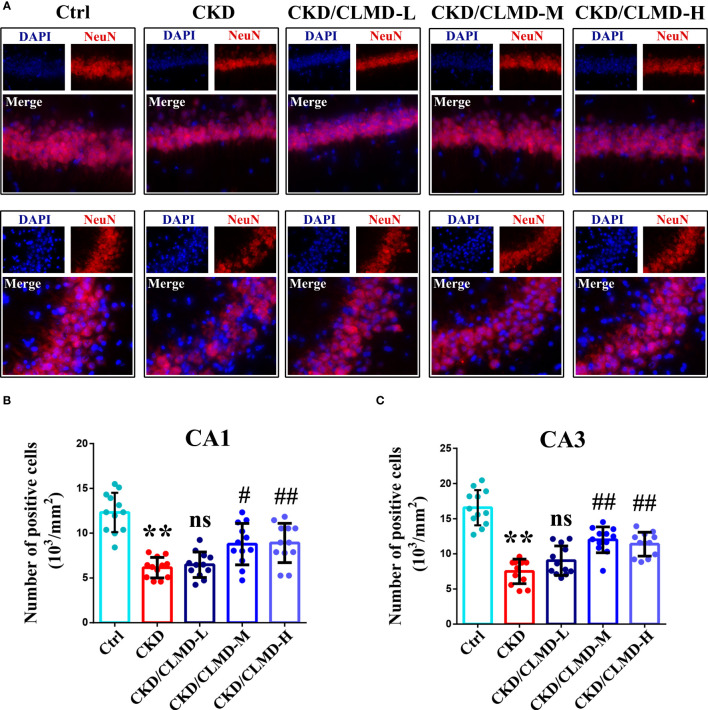
CLMD significantly reduced neuron loss in CKD mice. NeuN immunofluorescence staining of the CA1 and CA3 hippocampus region **(A)**. NeuN positive cell count graph of CA1 **(B)** and CA3 hippocampus region **(C)**. All values are expressed as individual plots with mean ± standard deviation (n = 4/group). P values versus control: **P < 0.01; P values versus CKD: #P < 0.05; ##P < 0.01; ns P > 0.05.

NeuN immunofluorescence staining was performed on each group of mice to evaluate the survival of neurons in the hippocampus. The results showed that the number of NeuN positive cells in the CA1 and CA3 regions of the hippocampus of CKD mice was significantly reduced, while doses of CLMD-M and CLMD-H could significantly reduce the number of neurons lost in the CA1 and CA3 regions of the hippocampus in CKD mice (**Figure 4**).

### 3.4 CLMD increased the expression of orexin, orexinR1, and orexinR2 in the hypothalamus of CKD mice

**Figure 5:**
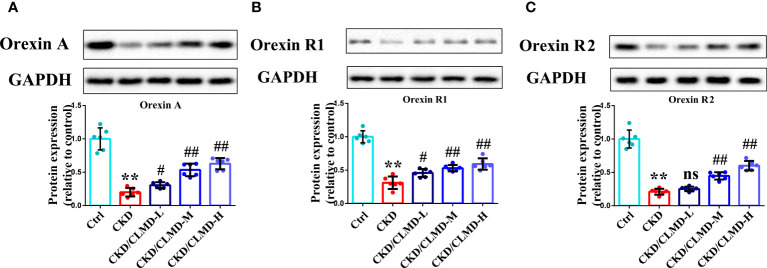
CLMD increased the expression of orexin, orexinR1, and orexinR2 in the hypothalamus of CKD mice. Representative western blot images of protein orexin-A **(A)**, orexin R1 **(B)**, and orexin R2 **(C)**, and the quantitative analysis of protein level of orexin-A, orexin R1, and orexin R2. All values are expressed as individual plots with mean ± standard deviation (n = 6/group). P values versus control: **P < 0.01; P values versus CKD: #P < 0.05; ##P < 0.01; ns P > 0.05.

Compared with the control group, the protein levels of orexin-A, orexin R1, and orexin R2 in CKD mice, induced by adenine, was significantly down-regulated (P<0.01). After intervention with CLMD, the level of orexin-A, orexin R1, and orexin R2 was partially restored. Compared with the CKD group, the protein levels of orexin-A, orexin R1, and orexin R2 in the CLMD treatment groups were significantly elevated (P<0.05 or P<0.01) and there was an obvious dose correlation. These results are shown in **Figure 5**.

### 3.5 CLMD activated the CaMKK2/AMPK pathway and inhibited the NF-κB pathway in the hippocampus

Compared with the control group, the protein levels of p-CaMKK2 and p-AMPK in CKD mice, induced by adenine, were significantly down-regulated (P<0.01), while the protein levels of p-NF-κB were significantly up-regulated.

**Figure 6:**
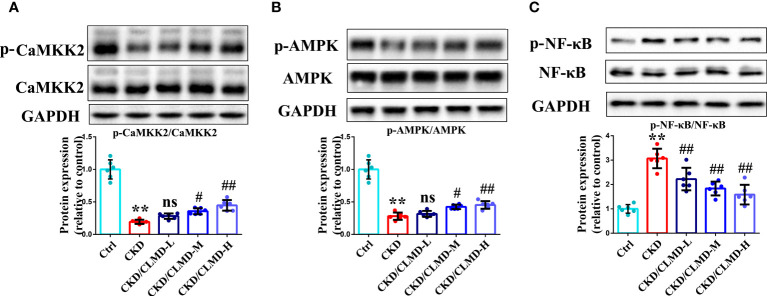
CLMD activated the CaMKK2/AMPK pathway and inhibited NF-κB pathway. Representative western blot images of protein p-CaMKK2 and CaMKK2 **(A)**, AMPK and p-AMPK **(B)**, NF-κB and p-NF-κB **(C)**, and the quantitative analysis of protein levels of p-CaMKK2/CaMKK2, p-AMPK/AMPK, and p-NF-κB/NF-κB. All values are expressed as individual plots with mean ± standard deviation (n = 6/group). P values versus control: **P < 0.01; P values versus CKD: #P < 0.05; ##P < 0.01; ns P > 0.05.

After intervention with CLMD, the levels of p-CaMKK2 and p-AMPK were partially restored. Compared with the CKD group, the protein levels of p-CaMKK2 and p-AMPK in the CLMD medium- and high-dose treatment groups were elevated significantly (P<0.05 or <0.01). Meanwhile, the phosphorylation level of NF-κB was significantly down-regulated by CLMD treatment (P<0.05 or P<0.01). These results are shown in **Figure 6**.

### 3.6 CLMD inhibited the expression of cytokines and up-regulated anti-inflammatory factors in the hypothalamus/hippocampus of CKD mice

**Figure 7:**
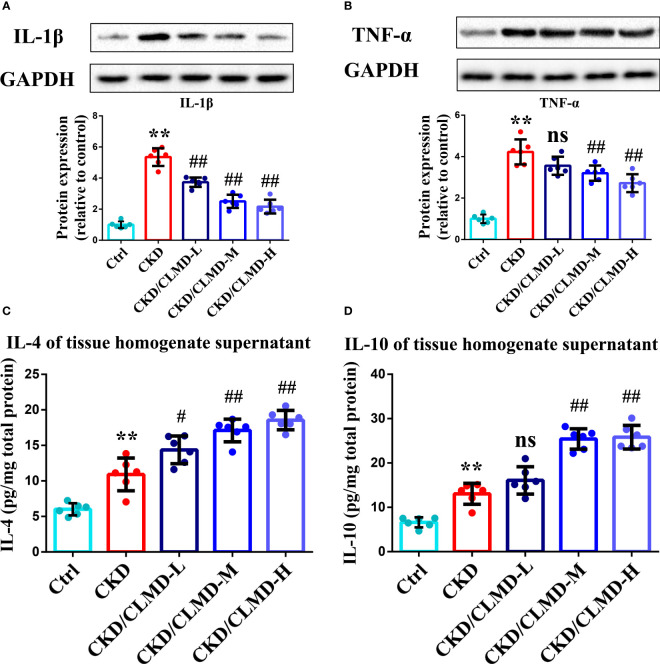
Representative western blot images of protein IL-1β **(A)**,TNF-α **(B)**, and the quantitative analysis of protein levels of IL-1β, TNF-α, and the quantitative analysis of hippocampal tissue anti-inflammatory factor IL-4 **(C)** and IL-10 **(D)**. All values are expressed as individual plots with mean ± standard deviation (n = 6/group). P values versus control: **P < 0.01; P values versus CKD: #P < 0.05; ##P < 0.01; ns P > 0.05.

To further assess potential alterations in the inflammatory responses in the brain after CLMD treatment, levels of cytokines in the hippocampus were determined by WB and ELISA. Cytokines included for analysis were IL-1β, TNF-α, IL-4, IL-6, and IL-10. Levels of IL-1β and TNF-α increased in the CKD group compared to that in the control group (P<0.01 for the hippocampus). After intervention with CLMD, the levels of IL-1β and TNF-α decreased significantly in a dose-dependent manner. Compared with the control group, levels of IL-4 and IL-10 increased significantly in the CKD group after CLMD treatment (P<0.01).The above results are shown in **Figure 7**.

### 3.7 CLMD decreased inflammatory cytokine levels and had no effect on creatinine and urea nitrogen in serum

**Figure 8:**
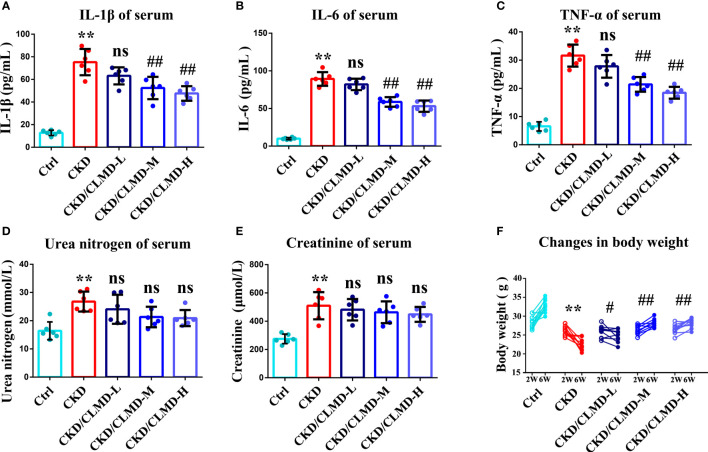
The quantitative analysis of serum anti-inflammatory factor IL-1β **(A)**, IL-6**(B)** and TNF-α **(C)**, and Serum Urea nitrogen **(D)** and Serum creatinine **(E)**, Change in body weight **(F)** All values are expressed as individual plots with mean ± standard deviation (n = 6/group). P values versus control: **P < 0.01 P values versus CKD: #P < 0.05; ##P < 0.01; ns P > 0.05.

We further tested whether CLMD could affect the peripheral inflammatory state of CKD mice. Preliminary inflammatory cytokines were determined using an ELISA. IL-1β, TNF-α, and IL-6 increased in the CKD group compared to that in the control group (P<0.01). After the administration of CLMD, these cytokines were significantly reduced in serum (**Figure 8**). In order to verify the safety of CLMD, we tested the serum creatinine and urea nitrogen levels in different group, and the results showed no statistical significance after CLMD treatment. These results are shown in **Figure 8**.

## 4 Discussion

Sleep disturbance is a common symptom in CKD patients and is a risk factor for the progression of CKD (18, 19). Reports suggest that about 50%–80% of patients experience insomnia or excessive daytime sleepiness (20, 21). Patients with CKD and end-stage renal disease have abnormal autonomic nervous regulation during normal awakening/sleep, and patients exhibit loss of parasympathetic excitability and reduced sympathetic hyper-excitability during the transition from the waking state to non-rapid eyes movement(REM) and REM sleep (22).

Chaihu-Longgu-Muli decoction (CLMD) is a well-used ancient formula for neuropsychiatric disorders, such as insomnia, anxiety, and dementia. To study whether CLMD could modulate CKD-related insomnia, we used a 0.25%-adenine diet to induce CKD in mice. After induction of CKD, the total activity of mice decreased at night-time, sleep latency increased, and sleep time decreased. After the administration of CLMD, nocturnal activity time increased, the number of activities increased, sleep latency decreased, and sleep time increased. Therefore, our results demonstrate that CLMD can improve sleep disturbances in adenine-induced CKD.

Further, we performed a preliminary exploration of the mechanisms of CLMD in improvement of sleep disorders. As a key modulator of sleep rhythm, decreases in orexin levels could shorten non-REM and REM sleep periods and increase wakefulness, and also cause anxiety. As mentioned earlier, previous studies have shown that the expression of orexin-A and orexin receptor-1 in the hypothalamus of CKD rats is low (12), indicating that orexin-A might be a therapeutic target of CKD-related insomnia. Here, compared with that in the control mice, the expression of orexin-A and orexin R1 in adenine-induced CKD mice was down-regulated, and after administration of CLMD, the levels of orexin-A, orexin R1, and orexin R2 were partially restored. This suggests that CLMD may improve the sleep rhythm of CKD mice by regulating the expression of orexin and orexin receptors in the hypothalamus.

Our research has also shown that CLMD has a protective effect on neurons while intervening to restore sleep rhythms in CKD mice. NeuN immunofluorescence detection on frozen sections of mouse brain showed that CLMD significantly reduced the loss of neurons in the CA1 and CA3 regions of the hippocampus in CKD mice. Further, results of the MWM task showed that after CLMD intervention, the learning and memory ability and spatial exploration ability of CKD mice were improved, and the cognitive function of CKD mice was also significantly improved.

Therefore, we further explored the effective mechanism of CLMD on cognitive improvement. It is currently known that persistent low-grade inflammation is considered to be an important part of CKD and an important cause of cardiovascular disease and other causes of death in patients. In a CKD cohort study, biomarkers of inflammation (IL-1β, IL-6, TNF-α, and other indicators) were negatively correlated with the measured values of renal function (23). While orexin-A regulates sleep and wakefulness, and at the same time acts as an anti-inflammatory neuropeptide to participate in the protection of neurodegeneration, it can also reduce neuroinflammation after intracerebral hemorrhage in mice through the OXR2/CaMKKβ/AMPK signaling pathway (24). At the same time, the combination of orexin-A and orexin receptors will inhibit the activation of NF-κB and participate in a neuro-protection function (7, 25).

Our results confirmed that the phosphorylation level of the CaMKK2/AMPK pathway in the hippocampus of CKD mice was significantly reduced and the phosphorylation level of NF-κB increased. CLMD intervention can significantly increase the phosphorylation level of CaMKK2/AMPK in the brain and inhibit the activation of NF-κB (**Figure 5**). At the same time, the levels of inflammatory factors in the hippocampus of CKD mice were detected. The results showed that the levels of TNF-α and IL-1β protein in the hippocampus of CKD mice increased, and the levels of anti-inflammatory factors, IL-4 and IL-10, decreased significantly. CLMD reduced the levels of TNF-α and IL-1β protein in the hippocampus of CKD mice, while increasing the levels of anti-inflammatory factors IL-10 and IL-4 (**Figure 6**). This indicates that CLMD could improve sleep by increasing the levels of orexin-A and orexin receptor-1 in CKD mice. Furthermore, CLMD could increase the phosphorylation levels of the CaMKK2/AMPK pathway in the hippocampus of CKD mice, inhibiting the activation of NF-κB and then down-regulating the release of pro-inflammatory factors in the brain to play a neuroprotective role.

We further tested whether CLMD could affect the peripheral inflammation state of CKD mice. The results showed the levels of IL-1β, IL-6, and TNF-α in the peripheral serum of CKD mice after CLMD intervention were also significantly reduced, further confirming that CLMD intervention effectively improved the chronic inflammatory state of CKD mice.

In order to verify the safety of CLMD, we tested the serum creatinine and urea nitrogen levels of CKD mice, and the results showed that not only did CLMD not increase the serum creatinine and urea nitrogen levels, but that renal function had a trend toward improvement after intervention with CLMD (no statistical significance). The comparison of the weight of mice before and after treatment also shows that CLMD intervention is beneficial to weight loss in mice in a CKD model, and this further confirms that CLMD is safe to apply to sleep disorders in CKD mice.

However, this study is just a preliminary pilot animal test, and more research is required to confirm the clinical significance of the study.

## 5 Conclusions

In summary, our results suggest that CLMD could improve sleep disorders and cognitive levels of CKD mice. The mechanism may be related to the up-regulation of orexin-A and increases in the phosphorylation level of CaMKK2/AMPK, which further inhibit NF-κB downstream signaling pathways without affecting renal function.

## Data availability statement

The original contributions presented in the study are included in the article/supplementary Files, further inquiries can be directed to the corresponding author/s.

## Ethics statement

The animal study was reviewed and approved by Animal Care and Use Institutional Committee of Chongqing Hospital of Traditional Chinese Medicine.

## Author contributions

FL and MS designed and conducted experiments. FL and X-LC participated in experiment design and article proof reading. G-BL assisted in data collection and manuscript preparation. X-MP, W-SD, K-ZW, X-LW, Y-YX and W-JX participated in experiments. MS performed data analysis. The final manuscript has been all authors read and approved by all authors.
